# Sick-listed persons’ experiences with taking part in an in-patient occupational rehabilitation program based on Acceptance and Commitment Therapy: a qualitative focus group interview study

**DOI:** 10.1186/s12913-015-1190-8

**Published:** 2015-11-27

**Authors:** Marit B. Rise, Sigmund Ø. Gismervik, Roar Johnsen, Marius S. Fimland

**Affiliations:** Department of Public Health and General Practice, Norwegian University of Science and Technology, Trondheim, Norway; Department of Physical Medicine and Rehabilitation, St. Olav’s University Hospital, Trondheim, Norway; Hysnes Rehabilitation Center, St. Olav’s University Hospital, Trondheim, Norway

**Keywords:** MeSH, Absenteeism, Occupational health, Rehabilitation, Return to work, Acceptance and commitment therapy, Cognitive behaviour therapy, Musculoskeletal diseases

## Abstract

**Background:**

Occupational medicine has shifted emphasis from disease treatment to disability rehabilitation and management. Hence, newly developed occupational rehabilitation programs are often generic and multicomponent, aiming to influence the sick-listed persons’ perception on return to work, and thereby support the return to work process. The aim of this study was to explore sick-listed persons’ experiences with taking part in an in-patient occupational rehabilitation program based on Acceptance and Commitment Therapy.

**Methods:**

Twenty-nine adults on sickness benefit or work assessment allowance due to musculoskeletal and/or common mental health disorders participated in this study. They were interviewed in focus groups at the beginning and at the end of a 3.5 week inpatient group-based occupational rehabilitation program in Central Norway. Key elements in the program were Acceptance and Commitment Therapy (ACT), physical exercise and creating a work-participation plan. The program was mainly group-based including participants with different diagnoses. Data was analyzed according to a phenomenological approach.

**Results:**

At the start of the program most participants expressed frustration regarding being sick-listed, external anticipations as well as hindrances towards returning to work, and described hope that the program would provide them with the skills and techniques necessary to cope with health problems and being able to return to work. At the end of the program the participants described that they had embarked upon a long process of increased awareness. This process encompassed four areas; an increased awareness of what was important in life, realizing the strain from external expectations and demands, a need to balance different aspects of life, and return to work as part of a long and complex process.

**Conclusions:**

The occupational rehabilitation program induced a perceived meaningful reorientation encompassing several aspects of life. However, the return to work process was described as diffuse and uncertain for most participants. The providers of occupational rehabilitation program should balance this reorientation with specific steps towards return to work. Effect studies and long-term qualitative studies evaluating how this affects long-term work- and health outcomes are underway.

## Background

Long-term sick-leave has considerable impact on the patients’ social functioning, on their families, the companies they work for, and for society [1]. Different steps have therefore been taken to prevent long-term sickness absence and permanent work disability. In many European countries legislation, as well as health, safety and environment acts have been implemented, and many different outpatient and inpatient rehabilitation programs have been established to facilitate return to work [2–5].

During the last decades, occupational medicine has shifted emphasis from disease treatment to disability rehabilitation and management [6, 7]. Long-term sickness-absence is now perceived as an interaction between the patient, the workplace, the health services, social surroundings, as well as financial aspects [6, 7]. Most programs designed to facilitate return to work for sick-listed patients have nevertheless been tailored for specific diagnoses. For example, several studies have investigated diagnose-specific return to work-interventions for musculoskeletal disorders [8–12]. Other studies have investigated occupational interventions for patients with mental health problems [13–16]. However, several studies have showed that many individuals are sick-listed due to several health problems and that co-morbidity is common [17–20]. We are therefore currently investigating a generic mainly group-based occupational rehabilitation program including patients with musculoskeletal-, mental- or unspecific disorders in the same group [21].

The multicomponent and primarily group-based rehabilitation program investigated in this study included three elements; Physical exercise, work-related problem solving, and psychological therapy. Physical exercise is intended to improve fitness, functional capacities and health, and to reduce fear of strain. Work-related problem solving aims to identify challenges and possibilities, and to create an individual return to work plan. The psychological approach is Acceptance and commitment therapy (ACT) [22], an approach which uses acceptance and mindfulness strategies, along with commitment and behaviour change strategies, to increase psychological flexibility. Psychological flexibility is described as “the ability to contact the present moment more fully as a conscious human being, and to change or persist in behaviour when doing so serves valued ends” [22], page 7. The goal is thus not to remove or “fix” problems, but to provide increased opportunities to live according to long-term desired qualities of life (in the following called values). Values are chosen by the individual and behavioural choices and committed actions are based on these values. ACT is increasingly used in various settings and for different patient groups [23] and has shown positive results in several areas [24–27]. However, only one study has investigated the utility of an ACT approach in the process of returning to work after sickness absence [28].

Previous research has shown that persons’ perceptions of illness, and their beliefs regarding the ability to recover or work, can be important predictors for return to work [29, 30]. The person’s expectations for recovery and future work are thus important factors in the return to work process [30–32]. An important question is how the occupational rehabilitation program investigated in this study may influence the participants’ perspective on their ability to cope with their health problems and the return to work process. The effect on future return to work is currently being investigated in an ongoing randomized controlled trial [21]. It seems plausible that physical exercise, creating a return to work plan, and ACTs intention to increase psychological flexibility and thereby coping skills, should change the participants’ perspective on their ability to cope with health problems in everyday life. It is also hypothesized that the occupational rehabilitation program would influence the sick-listed persons’ perspectives on returning to work, and that beliefs and confidence concerning return to work would be strong after taking part in the program. The aim of this study was therefore to explore sick-listed persons’ experiences with taking part in an in-patient occupational rehabilitation program based on ACT.

## Methods

This was a qualitative study based on focus group interviews with sick-listed patients participating in a 3.5 week inpatient group based occupational rehabilitation program in Central Norway. The study was nested into different quantitative studies, and all participants also took part in different quantitative studies [21, 33]. The interviews were conducted during winter 2012/2013.

### Participants and recruitment

Participants were invited to take part in focus group interviews at the day of arrival to the rehabilitation center. All participants in the rehabilitation program were invited to take part in the qualitative study. The participants were adults (18–60 years), on sick-leave for more than 8 weeks or receiving other temporary social security benefits after one year of sickness absence, due to musculoskeletal disorders, fatigue and/or common mental health disorders (stress, depression and anxiety). A few of the participants who were invited declined to participate in the first interviews due to other engagements at the time scheduled for the group interviews. At the time of the second interviews six of the participants who took part in the first interview declined to participate in a second interview. Six others wanted to take part in the second interview although they did not participate in the first.

A total of 29 persons participated in five focus groups, 23 persons at the start of the program and 23 at the end. Seventeen persons participated in both interviews, six persons participated only at the start of the program, and six persons only took part in interviews at the end. The sample consisted of 6 men and 23 women, a distribution which resembles the total group of participants at the rehabilitation center. The mean age was 44 years (range from 27–59 years). Their main diagnoses were musculoskeletal disorders including pain (18 participants), fatigue (9 participants) and mental health disorders (2 participants). The time they had been absent from working life varied from partly/fully sick-listed for 2–3 months to absent from working life several years.

### The rehabilitation program

This was a 3.5 week long (17 full work days) in-patient occupational rehabilitation program which took place at a rehabilitation center in Central Norway. The program was developed in collaboration between health personnel and the research group, and was piloted. The program was provided by interdisciplinary teams and in groups of 4-8 participants with different gender, age, and diagnoses. Each group was led by two coordinators who were responsible for the group sessions. The coordinators had various backgrounds, as physiotherapists, psychologists, social workers or nurses. They were all trained in Acceptance and Commitment Therapy (ACT) and were continuously supervised by a psychologist. Each participant was given one of these coordinators as their primary coordinator. The primary coordinator was responsible for the individual sessions. Acceptance and Commitment Therapy (ACT) and work related problems solving was provided in 16 h of group sessions and 5 h of individual consultations. In addition, the program included mindfulness sessions (3.5 h), psycho-educational group sessions (6.5 h), and one consultation with physician. Participants could also bring one or more persons (usually spouse, family member or close friend) to a “network day” with two group sessions (4 h) to enable next of kin to support behavioural changes after the program. The work related problem solving included the development of a return to work plan, where challenges and possibilities regarding the return to work process was identified and described. The goal was to motivate the participants to make realistic plans for increasing participation in working life. Supervised physical training was provided in group and individual sessions (a total of 12 h) and outdoors activities (1.5 h). In cooperation with the coordinator and the exercise coach each participant developed a personalized physical training plan. The program was based on the participants needs and wishes and the professionals’ clinical judgement. The main goal with the physical training was to increase strength and endurance and to reduce fear of movement. For further details about the program content, see previous publication [21].

### Data collection

Data was collected through focus group interviews. Focus groups were chosen as the data collection method since reflections in groups were a major element in the program, and we wanted to explore the participants’ experiences with discussions and interaction between the participants in the therapy group. The first interviews were conducted the first day of the rehabilitation program. Follow-up interviews were conducted the day before the end of the program – 3.5 weeks after the first interview. The participants were informed about the interviewers’ backgrounds and the main aim of the interviews; the participants’ expectations towards (first interview) and experiences with (second interview) taking part in the program. Interviews had no time limit, lasted between 45 and 90 min, and were based on an interview guide that included several topics we wanted to include in the interviews. This guide was slightly extended and modified during the data collection. Main themes were the participants’ experiences with health problems, work, and with being sick-listed, as well as their expectations towards the program and the potential changes during the rehabilitation (first interview). The second interview included the participants’ experiences with taking part in the program, perceived personal changes, and expectations for the future, including plans for return to work. Group discussions were encouraged during the interviews, as well as practical examples of the themes discussed. Four of the interviews were conducted by two researchers (SØG and MBR) and six by one (SØG). The interviewers discussed the main themes during the interview process and data was collected until data saturation [34]. When no new themes occurred in interviews data collection was stopped. Interviews were tape recorded and transcribed verbatim. Redundant words were omitted and local dialect was changed to written language. Quotes presented were translated from Norwegian to English by the first author (MBR).

### Data analysis

Data analysis was based on phenomenology and systematic text condensation [35]. Systematic text condensation encompasses the descriptive, more than interpretative, expressions of the participants in interviews [35]. A general data analysis for several research aims was conducted alongside the data collection. Large parts of the analysis (reading, discussion on overall impression, and initial coding) were conducted in a group of four persons with various backgrounds. Two of the authors are researchers and medical doctors (SØG and RJ). SØG is a specialist in rehabilitation of musculoskeletal disorders with competence in Acceptance and commitment therapy (ACT) and RJ has extensive experience as a general practitioner. MBR and MSF are public health researchers, MBR with background from psychology and MSF from exercise physiology. In addition, a fifth researcher (a psychiatrist familiar with ACT) took part in the initial stages of analysis. The main part of the analysis of the start interviews were conducted by the 1^st^ author (MBR).

Firstly, all interview transcripts were read to grasp the overall meaning of the data material, according to the process of systematic text condensation [35]. It became clear that the participants’ descriptions at the start were somewhat different from the descriptions given in the interviews at the end of the program. Main themes in the first interviews seemed to focus on the participants’ difficult situation due to health problems and sickness absence, such as “frustration” and “should have been back at work”, as well as “expectations to be helped by the rehabilitation program”. Examples of major themes in the interviews at the end of the program were “awareness process”, “my own values”, and “setting boundaries”. A detailed search for meaning units was conducted for the first interview and the second interviews separately. Meaning units are short or longer pieces of text which helps answering the research question [35]. The meaning units were then sorted, first into codes (summarizing what these meaning units jointly describe) and then into larger groups of codes. Examples of codes in the first interviews were “have to work”, “need a solution”, “to be sick-listed”. Examples from the second interviews were “more focus on values”, “expectations versus values”, and “uncertainty about future”. A synthesis of the code groups were written for the first and the second interviews separately. The themes from interviews at the start of the program were compared with the important themes at the end of the program, and differences between the two rounds of interviews were actively sought after. A full structural description of the findings was finally made and was discussed in the research group.

## Ethics

This study was approved of by the regional committee for medical and health research ethics in Central Norway (2012/856). The participants received oral and written information about the study and signed a consent form before taking part in interviews.

## Results

Findings from the interviews at the start of the program are presented first, and findings from interviews at the end of the program are presented afterwards. The main findings from the first and second interviews are described in Table 1.

**Table 1:** Main findings in start and end of program

Start of the program	End of the program
Frustration about being sick listed	*A process of increased awareness:*
External anticipations to return to work	- What is important to me?
Hindrances towards return to work	- Realizing the strain from external expectations and demands
Wanting a whole person approach and “a solution”	- A need to balance different aspects of life
	- Return to work as part of a long and complex process

Results are illustrated and elaborated by participant quotes, which are identified with gender and participant code, focus group number and time point.

### At the start of the program

#### Frustration about being sick listed

In all interviews at the start of the program the participants expressed frustration about being absent from work due to health problems. Many participants said that they should have been back at work by now and that they felt an obligation to return to work as soon as possible. Some participants said that they felt “stuck” in their health problems and described feelings of defeat due to not having succeeded to return.

The interviews at the start of the program included many expressions of a strong desire to be able to cope with health problems, and to function normally in the working-life. Most participants expressed that they wanted to return, some full time, some part time, and some in another job than their current employment. Several were unsure whether they would be able to return to work full time. A few of the participants who had been out of work for years doubted that they would be able to return to working life due to “having been absent too long”.

#### External anticipations to return to work

In addition to the feeling of obligation, participants also said that they experienced that people around them expected them to return to work, including employers, colleagues and more distant acquaintances. Many expressed that they were expected to work full time and to cope as normal members of society. Several said that nothing less than a 100–110 percent effort was accepted in today’s society and that fulfilling these demands was difficult.

*“The whole job market expects you to perform a 100 % all the time. You should make a lot of money… not everybody has the opportunity to do that. It seems like few workplaces are adapted to us who have challenges”.* (Female A, group 3, interview 1).

#### Hindrances towards return to work

The participants also described several hindrances towards returning to work. In addition to their health problems, such hindrances were employers and colleagues who did not understand the limitations embedded in the health problems. Some had experiences with employers who were unwilling to adjust the working situation to the participant’s needs.

*“It wasn’t my employer who was the problem to start with. But it ended up being the problem. The reason was a lack of understanding. I was very disappointed in the way he treated me after I started working 40 and 60 %. I felt that he was nagging. “When are you coming back?” “Are you working more next week?” “How long is your sickness-absence?” I felt a tremendous pressure and a lack of willingness to make workplace adjustment”. […].* (Female B, group 3, interview 1).

#### Wanting a whole person approach and “a solution”

Some participants described that previous treatments of their health problems had focused on the specific cause of sickness absence, such as a painful shoulder or a depression, and not the overall picture. The participants said that they hoped that the rehabilitation program would “include the whole of me”, and “the whole picture”. The participants expressed hope that the rehabilitation program would include techniques and advice that would help them to finally cope with their health problems and be able to return to work. This was described as “finding the key”, “learning the smart things to do”, or “to find the solution”.

*“I hope that they [the professionals] are knowledgeable and can teach me some techniques… I imagine that a lot can be done if I can learn some techniques… to change my mindset about thoughts and how to handle things”.* (Male A, group 5, interview 1).

### At the end of the program

#### A process of increased awareness

At the end of the program the participants said that taking part in the program first and foremost had started a process of increased awareness; of how their health problems had impacted their life and how they had, often unsuccessfully, tried to manage their problems in the past. Many described the program as “eye-opening” and as “a process of waking up”, and said that they had become aware of and realized several important things during the program.

*[…] “I have benefited from becoming more aware… I want to praise them [the professionals] for that… the awareness process. To make us aware of today, the society we live in, the stress, to calm down” […]* (Male B, group 3, interview 2).

The results showed that the process of increased awareness involved four themes:

#### What is important to me?

The participants described that they during the program had worked with personal aspects, such as what they found important in life. Some said that they had discovered personal desired qualities of life that they had not previously been aware of. Others said that they had re-discovered important qualities of life they had forgotten or neglected due to hard work and demanding lives. For many of the participants this increased awareness had been an emotional experience. When asked to describe her values, one participant referred to an exercise where she was asked to imagine her 90^th^ birthday party and what she wanted her family to say about her in a speech:

*“[My values are] to take care of myself and be present… for my children, that I can be present for them. We talked a lot about the 90 years birthday… “What do you want to hear?” […] And I would of course hear them say: “Yes, you have been a mother who always was there for us” and such things. And I am now, in a way. I am present, but it is something about being really present. Even though you are physically there, you don’t always feel that you are present”.* (Female C, group 4, interview 2)

According to the participants, they had realized that it was necessary to live according to personal values to be able to live a meaningful life. The participants described that such values included self-care to cope with health problems and stress, as well as activities which were important to them, such as having hobbies and spending time with family and friends. The participants said that long-term values, such as self-care, had become more important for them during the program, and that such values was necessary for them to pursue.

*“I need to focus on my values. I still have a long way to go. I have to think about what to do to ensure that I keep doing what is most important to me. Not the safe things, but focusing on the most important ones and stick to them. Maintain my exercise and do the things I should do instead of finding excuses. And I cannot take part in too many activities… I have to keep focus on where I’m going… to stick to my plan”.* (Female D, group 5, interview 2)

#### Realizing the strain from external expectations and demands

Already in the interviews at the start of the program the participants described experiences of expectations and demands from other people and from society, mainly to return to work and to work a 100 %. However, in the interviews at the end of the program the descriptions of expectations and demands also extended beyond the work sphere. At the end of the program many participants talked about expectations from family members, friends, neighbors, and more distant acquaintances, expectations regarding other areas than work. Common examples were expectancies regarding participation in social life and voluntary work, such as taking care of other people, bringing cakes to parties, and helping out in various projects.

*“The thing is that I have worked all the time. I haven’t had any time off. That’s how things are. You are helping out and taking responsibility for the children’s sports teams… handball, football, swimming, athletics… taking part everywhere. And that’s fine… it is your children, after all. But you end up taking care of others’ children too, so it becomes quite demanding”.* (Male C, group 4, interview 2)

Many described that they had come to realize that expectations and demands from others had impacted their lives considerably, and that constantly trying to fulfill other people’s expectations had made life very hard. The participants said that fulfilling these took a hard toll on important parts of life, often described as the important life values they had discovered or re-discovered during the program.

*[…] “It’s about being faithful to your values… values you might have set aside for many years. And having compromising on your values and what was good for you. You have to find your way back to the valuable stuff… and offer time and attention and nourishment… you might have forgot yourself in the middle of everything because life has been demanding. And you have let yourself become a pawn [in a game of chess]”.* (Female E, group 4, interview 2)

#### A need to balance different aspects of life

At the end of the program the participants talked a lot about achieving balance in life. Most of them described that some of the personal values they had discovered or re-discovered during the program was in conflict with expectations and demands from others, both within and outside the workplace.

*“We might be here [at the rehabilitation center] to learn to stop and to start seeing things from a new perspective… the way you view the world. What do you really want? Do you want to make a lot of money and be worn out when you are forty years old, or do you want to make a decent living and be able to live with yourself physically and mentally, and feel that you have the energy and time necessary to take care of people around you? I feel… my social life suffers… pals, friends and having the energy to take part in activities, instead of saying no. […] If you are absent from work one day someone can do your job, but nobody can take your place in your social life”.* (Male B, group 3, interview 2)

According to this, most participants had plans for achieving more balance between different aspects of life. Some described clear plans for behavioral changes, while other gave more diffuse descriptions, such as “living according to my values” and “setting boundaries”. Many of the participants said that the realization of how expectations and demands from others had impacted their lives had made them understand that it was necessary to set new boundaries. Boundaries included rejecting some requests and invitations, and to prioritize and choose between different activities. According to the participants, these measures would help balancing other people’s expectations and demands with self-care and other important life values.

*“I have learnt a lot of new things… new ways to handle things and to prioritize myself a little bit more instead of prioritizing everybody else. To start focusing on what you want for yourself and not only consider everybody else… at work or in private life”. […].*

*“One of my goals is to become good at saying «no». I often feel: «I don’t want to do this», but still I say yes. I am a world champion in offering to help even before anybody asks. I’m at least going to stop doing that”.* (Male A, group 6, interview 2)

#### Return to work as part of a long and complex process

At the end of the program most participants repeated and confirmed that they wanted to return to working life. But work was now described as one aspect of life which had to be balanced with other parts, such as self-care and time with family and friends. The “long process of increased awareness” the participants had started was described as encompassing all aspects of life, including return to work. In line with this, many of the participants said that they wanted to include important life values in the return to work process. Balancing different aspects of life was described as crucial to achieve sustainable return to work. In addition, many stated that they found it important to use the time necessary and gradually increase the amount of work, in order to avoid relapse to sickness-absence.

*[…] “when I’m going to find a job after I finish here [at the rehabilitation center] I will try to take some of this with me… the techniques and the perspectives on life. When I bring it along with me I will try to focus on… bring with me the thing about values, use them more and take the time to stop and think and not go back in the same pattern and the same pitfall… to keep working a lot”.* (Male B, group 3, interview 2)

Some of the participants said that they had come to realize that going back to work would take longer and be more difficult than they had anticipated before taking part in the program. Some explicitly expressed that they intended to pace themselves in the return to work process instead of rushing back.

As in the interviews at the start of the program, at the end of the program the participants expressed various degrees of hope and trust in their ability to return to work. A few of the participants described clear plans towards going back to work, such as making appointments with employer or social security officer, or to return part-time and subsequently increase the amount of work. Although many of the participants expressed that taking part in the program was of great personal significance, some said that the complexity of this awareness process made the overall future seem more diffuse than before the program. This diffuseness was also described in working life. For instance, some participants said that they found future working life more unresolved at the end of the program than before:

*“Male K: I am sick-listed and have two months left of it [sickness benefit]. When I came here my focus was to return to work when my absence period runs out. Now I have… realized that I might not be able to do that”.*

*Interviewer 2: Did the program change that?.*

*Male K: I might have realized that it [the back-pain problem] is more permanent and that it might not improve immediately.* (Male D, Group 1 + 2, interview 2).

A small group of participants, most of them long-term absent from working life, expressed that they had come to realize that they were not likely to return to work at all. Another small group discussed whether it was “worth sacrificing” self-care and other important qualities of life for full time work:

*“Now I am working 50 %. But I feel… I have always been working full time. So it is the same as not working and wanting to work a little. I have this urge to work more. But right before I came here and after I came here I have been thinking: “Why do I have to do that when it weighs down on so many other things.” Maybe I shouldn’t be working more and rather be thinking about the qualities of life related to other things than work. I have come to realize that work isn’t everything in life. Other things are more important and having the energy to do those are more important than spending all my energy at work. This has become clearer to me. I have become bold enough to dare saying that”.* (Female F, group 3, interview 2)

## Discussion

At the start of the program most participants expressed frustration regarding being sick-listed, external anticipations as well as hindrances towards returning to work, and described hope that the program would provide them with the skills and techniques necessary to cope with health problems and being able to return to work. At the end of the program the participants described that they had embarked upon a long process of increased awareness. This process encompassed four areas; an increased awareness of what was important in life, realizing the strain from external expectations and demands, a need to balance different aspects of life, and return to work as part of a long and complex process.

### Discussion of results

Most participants talked about discovering new personal values or re-discovering old ones. That persons participating in occupational rehabilitation appreciate being encouraged to focus on important personal values and needs has also been found elsewhere [36]. For instance, persons sick-listed due to mental health problems have described that reflecting on what is valuable in life and focus on life goals instead of symptoms, is necessary to be able to live a satisfactory life [37]. The results in the present study showed that the participants contrasted the more “internal” values with “external” expectations and demands from other people and society as a whole. The experiences of strong demands to function and work normally were during the program extended to life as a whole, and the participants had started the work to balance these external demands with personal values.

Many authors have described and discussed a balance between work on one hand, and non-professional parts of life on the other - often called the work-life-balance [38–40]. While work in pre-industrial times was closely linked to family and personal life, work after the eighteenth century became separate from “private” leisure time [41]. Authors have pointed to a “modern” division between private and public, as well as between life in the family/community and the engagement in work [41] (page 16). Although a new “merging” of work and private life might be seen due to technological development, we often think of work and leisure as two separate life arenas, where sick-leave is only possible in the work arena. In the present study, the participants did not divide their lives in work on the one hand and the rest of life on the other. Instead, they described incongruence, and a potential conflict, between external expectations/demands and internal personal values.

An important question when discussing the findings is whether the rehabilitation program has “highlighted” this conflict by transforming the initial sense of frustration and pressure to return to work into focusing on the meaning of life outside work. Virtanen and colleagues conducted an ethnographic study of sickness absence and whether absence could be understood as a practice, not only based on the individuals’ health, but also on the rules of the community they belonged to [42]. The authors argued that attitudes to work also were an expression of the attitudes which are rooted in the participants’ community. In the interviews at the start of the program, the participants focused on finding a solution to health problems and sickness absence. In accordance with the findings of Virtanen and colleagues’, one might ask whether the rehabilitation program has acted as a “community” which has communicated the importance of attending to desired qualities in life. Focusing on desired qualities of life is also in line with one of the main aspects of the ACT approach [23]. The program might thereby have induced a shift in perspectives on work. It is therefore reasonable to ask whether the program, including the rehabilitation therapists, have focused adequately on the return to work process.

Crooker and colleagues have similarly described that the balance between work and life outside work is influenced by the individual’s value system [40]. Values are described as a belief that “a specific mode of conduct or end state of existence is personally or socially preferable to an opposite or converse mode of conduct […]” (page 399). Crooker and colleagues further describe that the intensity of a value, i.e. how strongly held they are, might influence the experience of a balance with work and the rest of life. In addition, the authors describe that there might be congruence – or lack of congruence – between the values of the individual and members of different domains in this individual’s life. In the present study the newly discovered or re-discovered values seem to be incongruent with the external demands experienced by the participants. In light of Crooker and colleagues’ description of values, the participants’ long-term priorities seem to be strong, yet incongruent with the other values, in this case the society’s anticipation of them returning to work.

Previous studies have also found a conflict between desired qualities of life outside work and the values in the workplace, especially during the return to work process. In a study exploring Dutch women’s perceptions of work and sickness absence the women described a big gap between their realization of limitations in their work capacity and the expectations and demands at the workplace [43]. The recognition of the gap was attributed to the rehabilitation programs the women had participated in, a program that solely focused on the women’s personal work values without adjusting the work environment. As return to work is a complex process which extends beyond the individual and includes many different actors [44], working with the individual’s desired life values alone might not be sufficient for a successful return to work process [43].

The results in the present study showed that while work and absence from work was described as a major problem in the initial interviews, the focus in the follow-up interviews was on the process of awareness which the participants had been introduced to. Compared to the sense of pressure and frustration the participants expressed in initial interviews, most of the participants had achieved a more patient and somewhat “sober” attitude towards unrealistic expectations for a “quick fix” to return to work. Return to work was instead described as one of many aspects of a large, complex and more long-term process, and as one of several aspects in a multi-faceted view on life. This multi-faceted view of life is in line with previous research showing that participants in rehabilitation programs do not distinguish between work, family and the rest of life, but rather view them as parts of a whole [36]. This also corresponds with professionals’ perceptions of the return to work process. A Swedish study showed that professionals providing occupational rehabilitation programs perceived return to work as a long-term process which extended beyond the rehabilitation program [45].

Based on the results from the present study, a major question is whether Acceptance and Commitment Therapy, physical exercise and creating a return to work plan, as in this occupational rehabilitation program, is sufficient to induce sustainable return to work. A potential conflict between aiming to improve participants’ well-being and facilitating return to work has also been described previously [45]. In a Swedish study, health professionals working with occupational rehabilitation of patients with low-back pain emphasized that improving participants’ psychological and physiological well-being should in turn facilitate return to work, but that this sometimes conflicted with the intent to reinforce the return process [45]. A small qualitative study including women with stress-related health problems, who participated in a vocational rehabilitation program, showed that the participants found it easier to connect the experiences from the program to everyday life than to their job situations [46]. Changing approaches in the private arena was experienced as easier than changing behavior regarding work, and making concrete action in the job arena was difficult. Hellmann and colleagues argue that well-being and work are interconnected and influence each other, and that focusing on just one of the factors can hinder the return to work process [45, 46]. Haugli and colleagues interviewed persons with musculoskeletal and/or psychological health complaints who had taken part in an occupational rehabilitation program three years previously [36]. They found that self-understanding and viewing oneself as an active agent was perceived as necessary to be able to return to work. In addition to an increased focus on values and resources, this included changing the self-understanding to being able to take control over the rehabilitation process as well as the return to work process.

In light of the findings in the present study it would be reasonable to ask whether return to work presupposes a full reorientation of all parts of a person’s life, as well as whether this is a privilege reserved for generous welfare systems, as in Norway. Another important question would be whether this reorientation and self-realization process could take place while the person is working. Some studies have shown that returning to work might be beneficial for the worker’s mental health and function [47–50]. Authors have argued that training persons while they take part in normal working life might be beneficial compared to training them fully before entering the workplace [51]. The method of Individual Placement and Support has for instance shown health benefits for persons with severe mental health problems [52–55], and highlighted the benefits from being at work while struggling with health challenges.

The participants wanted to achieve a balance between different aspects in life, for instance between more external demands and expectations and the individual’s personal values. It thus seemed like all participants had a long-term goal of living “a good and balanced life” according to what they perceived as important in their lives. Holmgren and Ivanoff interviewed women who were sick-listed due to work-related strain, asking them about potential possibilities and obstacles for returning to work [56]. The women described that finding and doing meaningful self-chosen and enjoyable activities was a strategy to master everyday life. These findings are in line with the results of the present study, where the participants have (re) discovered and are connecting to personal values such as social contact.

However, Holmgren and Ivanoff also showed that formulating goals for the future and strategies for achieving these goals, as well as regaining faith in one’s own competence was described as necessary to return to work [56]. This resembles the concept of self-management of health problems, a concept that refers to how a person copes - or does not cope – with his or her health [57]. Self-management includes five core management or coping skills; Problem solving, decision-making, resource utilization, forming a patient/provider relationship, and taking action. Taking action includes for instance to make and carry out a short-term action plan. While participants in the present study had made different action plans regarding several aspects of their life, few had made concrete work-related steps of action. Resembling the model of self-management, the Stages of change model describes how different stages in the change process influence the motivation, readiness, and capability of making behavioral changes [58]. The Action stage in this model is the stage where persons are no longer contemplating whether to make behavioral changes but is taking concrete steps in modifying a behavior. The results from the present study indicate that the program has helped the participants to investigate and assess what they find important in life. It is however unclear whether the participants have achieved the ability to transform these personal values into concrete steps in the direction towards return to work. The results from this study are relevant for rehabilitation programs where the overarching goal is to improve the return to work process and eventually return to working life. The act of balancing the focus on exploring personal values with specific plans supporting return to work is a challenge for professionals working in occupational rehabilitation facilities.

### Strengths and limitations

This is a quite large qualitative study including a total of 29 participants. Although the proportion of women versus men in the study sample resembles the participants at this occupational rehabilitation center, the results might not be representative for other groups of sick-listed persons. Large parts of the data collection were conducted by two researchers, one medical doctor with clinical experience from occupational rehabilitation and one public health researcher. This provided various preconceptions and thus strengthened the data collection. Data analysis was conducted in a group including all four authors, two researchers with medical background and two researchers within social science/public health. In addition, a third researcher with medical background (psychiatrist) participated in the initial analysis. The findings were discussed in the group to strengthen diversity during the interpretations of the data and in affirmation of the findings.

There are also some potential limitations. The sample of participants who took part in the interviews at the start of the program was not identical with the sample at the end. Six persons just participated in the first interview, while six just took part at the end. This might have influenced the results and the changes in perspectives during the program.

Focus groups use the group interaction to create discussions and thereby explore important themes, as well as revealing values or norms in the group [59]. Individual interviews do not include discussions and interactions, but can be advantageous in exploring personal experiences and changes. The focus group interviews were conducted in the same groups that had worked together during the rehabilitation program. This was chosen since the participants already were familiar and we assumed that this would lead to spirited discussions. On the other hand, interviewing the participants in their therapy groups could have reduced the possibility for capturing any negative experiences. The participants might have had negative experiences with the program or with the group work in particular, which they did not feel comfortable voicing in these groups.

One medical doctor (SØG – conducted all interviews) and one researcher within public health (MBR – participated in four interviews) conducted the interviews. The choice of interviewers might have influenced the data collection by focusing more on the awareness process and less on return to work, possibly influencing the results.

It is important to keep in mind that the follow-up interviews were conducted at the end of the program - only 3.5 weeks after the rehabilitation program started. This time frame might not have been sufficient to allow for the necessary changes to have taken place, especially regarding return to work. Interviewing the participants at the end of the program did thus not allow for experiences after returning home from the rehabilitation center. More long-term exploration of the experiences after the program was beyond the sphere of this study, but would provide important indications on the following return to work process.

## Conclusion

This study explored sick-listed persons’ experiences with taking part in an in-patient occupational rehabilitation program based on Acceptance and Commitment Therapy.

At the end of the program the participants described that they had embarked upon a long process of increased awareness, acknowledging personal values and achieving balance in life. Although many participants had made important changes in attitudes and behavior, few had made concrete action-plans regarding return to work. The providers of occupational rehabilitation program should balance this reorientation with specific steps towards return to work. It would be important to discuss whether a total reorientation encompassing all aspects of life is needed to achieve sustainable return to work. Effect studies from rehabilitation programs and long-term qualitative studies on participants’ perceptions on return to work are undertaken to fully evaluate outcomes from occupational rehabilitation.

## Abbreviation

ACT: acceptance and commitment therapy
